# Multiple gene sequencing for risk assessment in patients with early-onset or familial breast cancer

**DOI:** 10.18632/oncotarget.7027

**Published:** 2016-01-27

**Authors:** Po-Han Lin, Wen-Hung Kuo, Ai-Chu Huang, Yen-Shen Lu, Ching-Hung Lin, Sung-Hsin Kuo, Ming-Yang Wang, Chun-Yu Liu, Fiona Tsui-Fen Cheng, Ming-Hsin Yeh, Huei-Ying Li, Yu-Hsuan Yang, Yu-Hua Hsu, Sheng-Chih Fan, Long-Yuan Li, Sung-Liang Yu, King-Jen Chang, Pei-Lung Chen, Yen-Hsuan Ni, Chiun-Sheng Huang

**Affiliations:** ^1^ Graduate Institute of Clinical Medical Science, China Medical University, Taichung, Taiwan; ^2^ Department of Medical Genetics, National Taiwan University Hospital, Taipei, Taiwan; ^3^ Department of Surgery, National Taiwan University Hospital, Taipei, Taiwan; ^4^ Department of Medical Oncology, National Taiwan University Hospital, Taipei, Taiwan; ^5^ Department of Pediatrics, National Taiwan University Hospital, Taipei, Taiwan; ^6^ Division of Medical Oncology, Department of Oncology, Taipei Veterans General Hospital, Taipei, Taiwan; ^7^ Department of Surgery, Shin Kong Wu Ho-Su Memorial Hospital, Taipei, Taiwan; ^8^ Department of Surgery, Chung Shan Medical University Hospital, Taichung, Taiwan; ^9^ Graduate Institute of Cancer Biology, China Medical University, Taichung, Taiwan; ^10^ Center of Genomic Medicine, National Taiwan University, Taipei, Taiwan; ^11^ Department of Surgery, Cheng Ching Hospital, Taichung, Taiwan

**Keywords:** multiple gene sequencing, hereditary breast cancer, BRCA, variant of uncertain significance, genetic counseling

## Abstract

Since *BRCA* mutations are only responsible for 10–20% of cases of breast cancer in patients with early-onset or a family history and since next-generation sequencing technology allows the simultaneous sequencing of a large number of target genes, testing for multiple cancer-predisposing genes is now being considered, but its significance in clinical practice remains unclear. We then developed a sequencing panel containing 68 genes that had cancer risk association for patients with early-onset or familial breast cancer. A total of 133 patients were enrolled and 30 (22.6%) were found to carry germline deleterious mutations, 9 in *BRCA1*, 11 in *BRCA2*, 2 in *RAD50*, 2 in *TP53* and one each in *ATM, BRIP1, FANCI, MSH2, MUTYH*, and *RAD51C*. Triple-negative breast cancer (TNBC) was associated with the highest mutation rate (45.5%, *p* = 0.025). Seven of the 9 *BRCA1* mutations and the single *FANCI* mutation were in the TNBC group; 9 of the 11 *BRCA2*, 1 of the 2 *RAD50* as well as *BRIP1*, *MSH2, MUTYH*, and *RAD51C* mutations were in the hormone receptor (HR)(+)Her2(−) group, and the other *RAD50, ATM*, and *TP53* mutations were in the HR(+)Her2(+) group. Mutation carriers were considered as high-risk to develop malignancy and advised to receive cancer screening. Screening protocols of non-*BRCA* genes were based on their biologic functions; for example, patients carrying *RAD51C* mutation received a screening protocol similar to that for *BRCA*, since *BRCA* and *RAD51C* are both involved in homologous recombination. In conclusion, we consider that multiple gene sequencing in cancer risk assessment is clinically valuable.

## INTRODUCTION

Breast cancer is the leading cause of cancer-related death in women worldwide [1]. Previous twin studies suggest that 12–30% of breast cancers are primarily genetic in origin and result from autosomal dominant inheritance of a single gene mutation [2, 3]. The best-known genes are *BRCA1* and *BRCA2*, which cause hereditary breast and ovarian cancer syndrome (HBOC). Genetic counseling and a *BRCA* gene test is recommended for breast cancer patients with early-onset or a significant family history. This strategy significantly reduces cancer-related mortality in *BRCA* mutation carriers, who receive regular cancer screening or prophylactic mastectomy and oophorectomy. However, pathogenic mutations of *BRCA1* and *BRCA2* only explain 10–20% of breast cancers in patients with early-onset or a significant family history. Of the non-*BRCA* genes, *ATM*, *BRIP1*, *PALB2*, *PTEN* and *CHEK2*, are reported to be medium-to-high penetrance genes that cause hereditary breast cancer [4–6]. A longitudinal study showed that, by 70 years of age, the absolute breast-cancer risk for female *PALB2* mutation carriers ranges from 33% (95% CI, 25 to 44) for those with no family history of breast cancer to 58% (95% CI, 50 to 66) for those with a family history [7]. Comprehensive multiple gene sequencing is therefore necessary to understand the predisposing genetic factors in development of breast cancer.

Next-generation sequencing (NGS) technology makes it possible to sequence large numbers of target genes and is now used not only in research, but for multiple gene testing for clinical application. Although multiple predisposing genes can be sequenced in parallel, several points have not been answered about the application of it into clinical practice. First, it is not known how many predisposing genes need to be tested in these patients and whether there is an association between gene and tumor phenotype (pathology). Second, there is no consensus on the best approach to genetic counseling, cancer-risk assessment, and intervention for patients with non-*BRCA* mutations. Third, it is difficult to distinguish genetic variants of uncertain significance (VUS), especially for non-*BRCA1/2* predisposing genes, in clinical patients.

In this study, we developed a customized sequencing panel containing 68 genes with a known and potential association with hereditary cancer syndromes (Table 1). Using this panel, we aimed to assess the clinical value of multiple predisposing genes in breast cancer patients with an early-onset or a significant family history.

**Table 1:** List of genes for sequencing and reasons for their inclusion

Gene	Hereditary syndrome or increased breast cancer risk (BC risk↑)	DNA repair	Gene	Hereditary syndrome or increased breast cancer risk (BC risk↑)	DNA repair
*APC*	FAP		*GT198*	BC risk↑	
*ARLTS1*	BC risk↑		*ku70*		NHEJ
*ATM*	BC risk↑		*ku80/XRCC5*	BC risk↑	NHEJ
*BARD1*	BC risk↑		*MAP3K1*	BC risk↑	
*BMPR1A*	JPS		*MDM4*	BC risk↑	
*BRCA1*	HBOC		*MLH1*	Lynch syndrome	MMR
*CDH1*	Gastric/breast cancer		*MLH3*	Lynch syndrome	MMR
*CHEK2*	BC risk↑		*MRE11*	BC risk↑	HR, NHEJ
*DDB1/XPE*	CS	NER	*MSH2*	Lynch syndrome	MMR
*DDB2/XPE*	CS	NER	*MSH3*	Lynch syndrome	MMR
*EPCAM*	Lynch syndrome		*MSH6*	Lynch syndrome	MMR
*ERCC1*	XP	NER	*MUTYH*	MYH-polyposis	BER
*ERCC2/XPD*	XP, CS, TTD	NER	*NBN*	BC risk↑	
*ERCC3/XPB*	XP, CS, TTD	NER	*NBS1*	BC risk↑	HR, NHEJ
*ERCC4*	XP	NER	*OGG1*	BC risk↑	BER
*ERCC5*	XP, CS	NER	*PMS1*	Lynch syndrome	MMR
*ERCC6/CSB*	CS,	NER	*PMS2*	Lynch syndrome	MMR
*ERCC8/CSA*	CS	NER	*polymerase delta1*		TLS
*FANCA*	Fanconi anemia	HR	*polymerase epsilon*		TLS
*FANCB*	Fanconi anemia	HR	*polymerase beta*		TLS
*FANCC*	Fanconi anemia	HR	*polymerase eta*		TLS
*FANCD1/BRCA2*	Fanconi anemia, HBOC	HR	*polymerase kappa*		TLS
*FANCD2*	Fanconi anemia	HR	*PTEN*	Cowden syndrome	
*FANCE*	Fanconi anemia	HR	*RAD50*	BC risk↑	HR, NHEJ
*FANCF*	Fanconi anemia	HR	*RAD51*	BC risk↑	HR
*FANCG/XRCC9*	Fanconi anemia	HR	*RAD51D*	BC risk↑	HR
*FANCI*	Fanconi anemia	HR	*SMAD4*	JPS	
*FANCJ/BRIP1*	Fanconi anemia, BC risk↑	HR	*STK11*	Peutz-Jeghers syndrome	
*FANCL/PHF9*	Fanconi anemia	HR	*TP53*	Li frenmanii	
*FANCM*	Fanconi anemia	HR	*XPA*	XP	NER
*FANCN/PALB2*	Fanconi anemia, BC risk↑	HR	*XPC*	XP, CS, TTD	NER
*FANCO/RAD51C*	Fanconi anemia, BC risk↑	HR	*XRCC2*	BC risk↑	NHEJ
*FANCP/SLX4*	Fanconi anemia, BC risk↑	HR	*XRCC3*	BC risk↑	NHEJ
*FGFR2*	BC risk↑		*XRCC4*	BC risk↑	NHEJ

CS: Cockayne syndrome; FAP: familial adenomatous polyposis; HBOC: hereditary breast and ovarian cancer syndrome; JPS: juvenile polyposis syndrome; TTD: trichothiodystrophy; XP: xeroderma pigmentosum; BER: base excision repair; HR: homologous recombination; NER: nucleotide excision repair; NHEJ: nonhomologous DNA end joining; MMR: mismatch repair; TLS: translesion synthesis

## RESULTS

### Patients' characteristics and performance of the illumina DNA sequencing

A total of 133 breast cancer patients were enrolled in this study. Their median age at diagnosis was 44 years; 41 were aged £ 35 years, 56 were 35–50 years, and 36 were > 50 years. Thirteen patients had metachronous breast cancer, five had a history of ovarian cancer, two had a history of colon cancer, and one each with a history of gastric cancer, nasopharyngeal cancer and multiple myeloma. A family history of breast cancer, ovarian cancer, prostate cancer, male breast cancer, or other malignancies was found in 97, 15, 7, 2, and 42 patients, respectively. The clinical characteristics of the patients are listed in Table 2.

**Table 2:** Characteristics of the study participants and comparison of patients with and without a pathogenic mutation

Variants	Without	With	*P* value
Patient number	103	30	
Median (range)	44 (25–75)	41 (29–60)	0.309
≤ 35 years (patient no.)	30	11	
> 35–50 years (patient no.)	45	11	
> 50 years (patient no.)	28	8	
Personal history			
Single/Metachronous breast cancer	94/9	26/4	0.456
Ovarian cancer	4	1	
Other cancer	4	1	
Molecular type			0.025
HR(+)Her2(−)	68	16	
HR(+)Her2(+)	16	4	
HR(−)Her2(+)	7	0	
TNBC	12	10	
Family cancer history			
Breast cancer	74	23	0.650
Ovarian cancer	10	5	0.327
Prostate cancer	3	4	0.024
Male breast cancer	0	2	0.008
Other cancers	31	11	0.510
Criteria of enrollment			0.304
(1)	23	6	
(2)	45	10	
(3)	34	15	

(1) Early-onset breast cancer (age ≤ 35 years) or bilateral breast cancer (without family hsitory)

(2) Breast cancer onset age ≤ 50 years and at least one first or second-degree relative with breast cancer or ovarian cancer

(3) Breast cancer onset after the age of 50 years, but with two relatives with breast cancer or one with ovarian cancer.

The average mean depth in the coding exons of the 68 genes was 195X (range: 2–348), respectively. The coding sequencing exons covered by at least 50 reads were 90.8% (range of depth per sample: 82.0%–94.5%).

### Deleterious mutations

**Figure 1:**
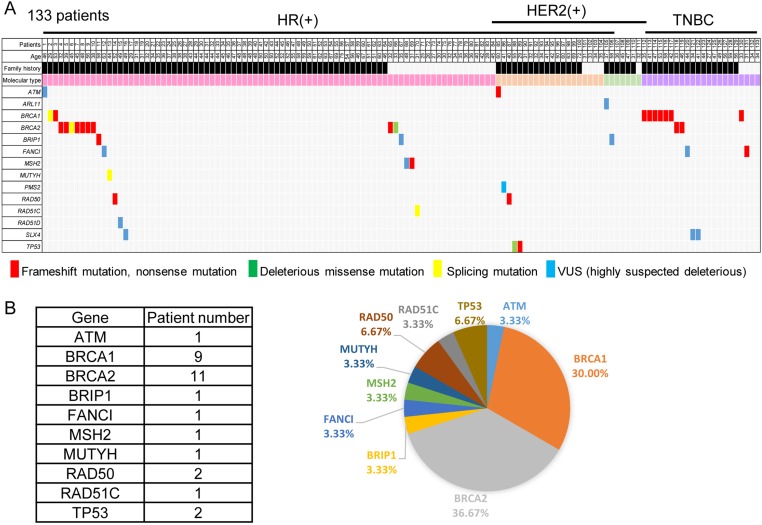
Mutation of predisposing genes in breast cancer patients with early-onset or a family history (**A**) Each of the predisposing genes identified in the patients is listed on the left. A family history is shown in black. For the molecular types, HR(+)Her2(−) breast cancer is colored pink, HR(+)Her2(+) orange, HR(−)Her2(+) light green, and TNBC purple. (**B**) Twenty-five mutations of a predisposing gene were identified, 1 (4.0%) in *ATM*, 8 (32.0%) in *BRCA1*, 10 (40.0%) in *BRCA2*, and 1 each (4.0%) in *BRIP1*, *FANCI*, *MSH2*, *RAD50*, *RAD51C*, and *TP53*.

As shown in Figure 1A and 1B, 30 patients (22.6%) were found to have germline heterozygous deleterious mutations of known cancer susceptibility genes, 9 in *BRCA1*, 11 in *BRCA2*, 2 in *RAD50*, 2 in *TP53* and one each in *ATM*, *BRIP1*, *FANCI*, *MSH2*, *MUTYH*, and *RAD51C*. The mutation prevalence for *BRCA1* and *BRCA2* in this cohort was 15.0%, indicating multiple gene sequencing increasing about 7.5% of the detection rate.

As shown in Table 3, analysis of mutation type showed that nonsense mutations were found in 8 patients, frameshift mutations in 15, missense mutations in 3, and mutations involving uncorrected splicing in 4. All of the nonsense and frameshift mutations were located in exons. One patient had a missense mutation of BRCA2 p.G2748D, which is reported to result in defective homologous recombination [8, 9], while others carried the heterozygous TP53 p.G245S or p.R248Q mutation, which result in a defective function of TP53 protein [10]. The intronic deletion of chr17:.g.41251910_41251919delGTAAAGAACA leads to deletion of a branch site in *BRCA1* intron 6 [11], while the *BRCA2* c.G631C mutation affects the donor site for splicing and the *RAD51C* c.905–2A > C and *MUTYH* c.934–2A > G mutation affects the recipient site; these three mutations, each found in 1 patient, are considered to cause uncorrected splicing and to be deleterious.

**Table 3:** Deleterious mutations identified in this cohort

Gene	Mutation	Transcript	gDNA/cDNA	Amino acid	Reported/novel
*ATM*	frameshift deletion	NM_000051	c.8434_8435delTC	p.2812del	Novel
*BRCA1*	frameshift deletion	NM_007294	c.1934delC	p.S645fs	Novel
*BRCA1*	frameshift deletion	NM_007294	c.1361delG	p.S454fs	Novel
*BRCA1*	frameshift deletion	NM_007294	c.470_471delCT	p.S157fs	Reported, *rs80357887*
*BRCA1*	splicing	NG_005905	g.41251910_41251919delGTAAAGAACA		Reported [30]
*BRCA1*	frameshift deletion	NM_007294	c.5470_5477delATTG GGCA	p.I1824Dfs	Reported, *rs80357973*
*BRCA1*	frameshift deletion	NM_007294	c.3770_3771delAG	p.E1257Gfs	Reported, *rs80357993*
*BRCA1*	frameshift deletion	NM_007294	c.3228_3229delAG	p.G1077Afs	Reported, rs80357635
*BRCA1*	nonsense mutation	NM_007294	c.3607C > T	p.R1203Ter	Reported, *rs62625308*
*BRCA1*	frameshift deletion	NM_007294	c.2679_2682delGAAA	p.K893fs	Reported, *rs80357596*
*BRCA2*	nonsense mutation	NM_000059	c.8934delA	p.E2981KfsTer7	Novel
*BRCA2*	nonsense mutation	NM_000059	c.6645delC	pS2216PfsTer13	Novel
*BRCA2*	nonsense mutation	NM_000059	c.5574_5577delAATT	p.I1859KfsTer3	Novel
*BRCA2*	nonsense mutation	NM_000059	c.5164_5165delAG	p.S1722YfsTer4	Reported, *rs80359490*
*BRCA2*	missense mutation	NM_000059	c.G8243A	p.G2748D	Reported, *rs80359071*
*BRCA2*	splicing	NM_000059	c.G631C		Reported, *rs80358871*
*BRCA2*	frameshift deletion	NM_000059	c.6448delA	p.K2150fs	Novel
*BRCA2*	frameshift deletion	NM_000059	c.2806_2809del	p.A938Pfs	Reported, *rs80359351*
*BRCA2*	frameshift deletion	NM_000059	c.8531_8532del	p.E2844fs	Novel
*BRCA2*	frameshift insertion	NM_000059	c.7407dupT	p.T2469fs	Novel
*BRCA2*	frameshift deletion	NM_000059	c.8323delA	p.M2775CfsTer2	Novel
*BRIP1*	nonsense mutation	NM_032043	c.G1343A	p.W448X	Novel
*FANCI*	nonsense mutation	NM_001113378	c.G568T	p.E190X	Novel
*MSH2*	nonsense mutation	NM_000251	c.C2785T	p.R929X	Reported,
*MUTYH*	splicing	NM_001128425	c.934–2A > G		Reported, *rs77542170*
*RAD50*	frameshift insertion	NM_005732	c.2157dupA	p.L719fs	Novel
*RAD50*	frameshift deletion	NM_005732	c.2498_2499del	p.Q833fs	Novel
*RAD51C*	splicing	NM_058216	c.905–2A > C		Novel
*TP53*	missense mutation	NM_000546.5	c.733G > A	p.G245S	Reported, *rs28934575*
*TP53*	missense mutation	NM_000546.5	c.743G > A	p.R248Q	Reported, *rs11540652*

### Missense mutations and variants of uncertain significance

A total of 14717 missense mutations were identified among the 68 genes in the 133 patients. After searching for the database (http://www.ncbi.nlm.nih.gov/snp/) and bioinformatics analyses to evaluate the pathogenicity, most of the missense mutations were considered as benign variants and 12 missense mutations were classified as VUS with suspicion of being deleterious, averaging 0.09 VUS per participant (Table 4).

**Table 4:** Variants of uncertain significance strongly suspected of being deleterious mutations

gene	*rs* number	cDNA position	amino acid	ESP6500	1000 Genomes	Patient number	Polyphen2	SIFT	GVGD align
*ARL11*	-	C467T	A156V	-	-	1	1	0	-
*ATM*	-	A8450G	Y2817C	-	-	1	1	0.004	C65
*BRIP1*	-	A2324G	N775S	-	-	1	1	0	-
*BRIP1*	*rs201869624*	C2440T	R814C	-	0.0005	1	1	0.019	-
*FANCI*	*rs149243307*	G286A	E96K	0.000154	0.00279553	2	1	0.004	-
*MSH2*	-	G1601T	R534L	-	-	1	1	0	C65
*PMS2*	rs182246929	C883T	R295W	-	0.000199681	1	1	0	C65
*RAD51D*	*rs145309168*	T932A	I311N	0.000231	0.0009	1	0.998	0	-
*SLX4*	-	G1457A	R486H	-	-	1	1	0	-
*SLX4*	-	T2453C	L818P	-	-	1	1	0	-
*SLX4*	*rs201622632*	A2381T	D794V	-	0.000199681	1	0.993	0	

**Figure 2:**
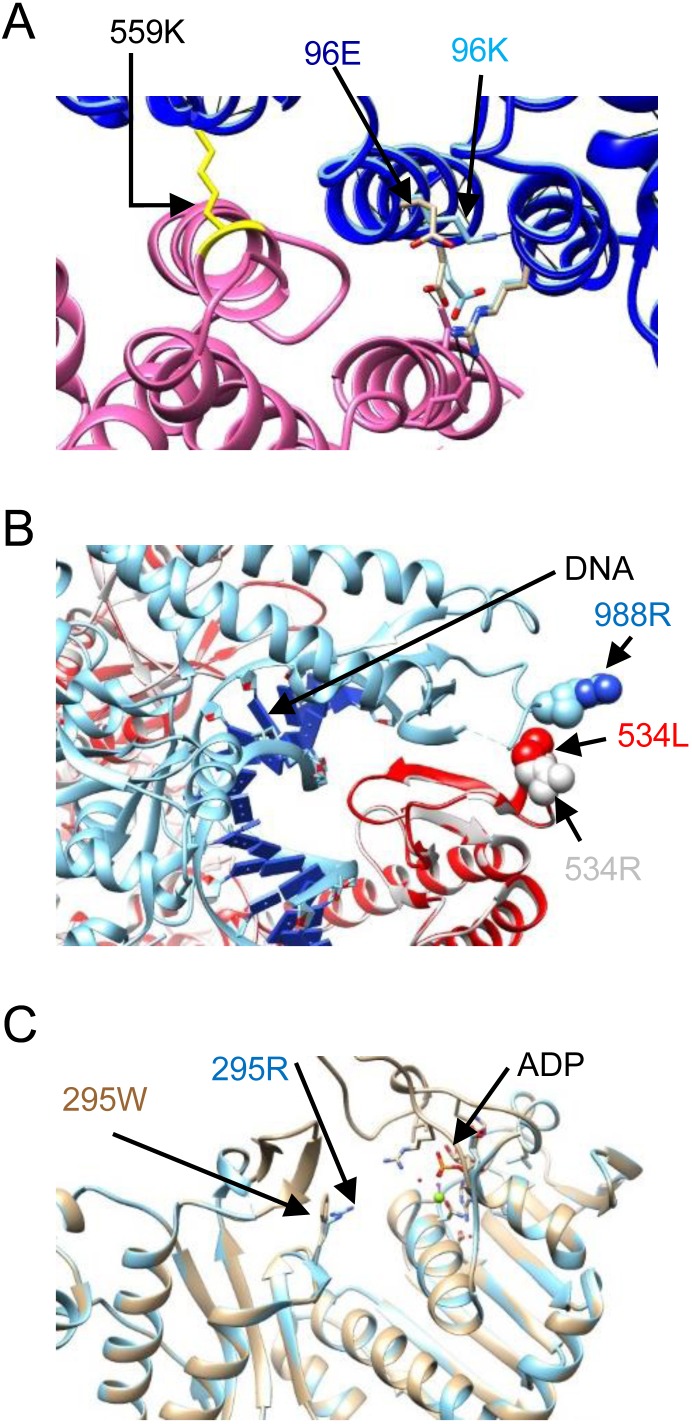
Structural analyses of three mutations (**A**) Ribbon presentation of the FANCI E96K mutant structure (dark blue) superimposed on the structure of the wild type FANCI interacting with FANCD2 (FANCI, light blue; FANCD2, purple; pdb 3S4W) to form the ssDNA groove. This interface is maintained by Van Der Walls forces between FANCD2 HD2 (Leu614) and FANCI solenoid 1 (Leu92, Met94, and Leu95). The lysine substitution, with a longer sidechain, may disrupt the FANCI cap-solenoid 1 structure, leading to disturbance of the binding affinity for FANCD2 and influencing the ssDNA groove. This may result in defective function of the FANCI-FANCD2 complex and inability to carry out DNA interstrand cross-linking. FANCD2 Lys559 (yellow) is a mono-ubiquitination site. (**B**) Ribbon presentation of the MSH2 R534L structure (red) superimposed on that for the wild type MSH2-MSH6 complex (pdb: 2O8E, light gray; MSH6, light blue; DNA helix, dark blue), showing that MSH2 Arg534 is located at the clamping region, which is involved in DNA contact and matching of MSH6. Substitution of the arginine with leucine reduces the basic nature of this region, which may alter the attraction of DNA. This structure change also affects the interaction with MSH6. MSH2 p. R534L is highly suspected to have decreased function. (**C**) PMS2 p. R295W (khaki color) superimposed on the wild type PMS2 structure (pdb: 1EA6, light blue). This missense mutation changes the polar amino acid arginine to the non-polar tryptophan and is located nearby the entrance to the ATP binding pocket. However, whether this causes a functional defect requires further functional assays.

For variants identified by above programs, we search for the protein database to simulate the mutant structure for visually checking the potential deleterious impact to the protein. Three missense mutations, FANCI p.E96K, MSH2 p.R534L, and PMS2 p.R295W, were further evaluated by simulation using known protein templates [12–14]. As shown in Figure 2 and Supplementary Figure 4, the missense mutation FANCD2 E96K may disrupt the FANCI-FANCD2 complex and inability to carry out DNA interstrand cross-linking (Figure 2A and Supplementary Figure 4A and 4B). The MSH2 p. R534L may affect the mismatch repair by influencing the DNA attraction and interaction with MSH6 protein. The other missense mutation PMS2 p. R295W changes the polarity of the amino acid position 295 and may affect the ATP entry to the ATP binding pocket. However, whether these causes a functional defect requires further functional assays.

### Association between genetic mutations and clinicopathologic characteristics

The mutation prevalence was 26.8% in the group aged £ 35 years, 19.6% in the group aged 35–50 years, and 22.2% in the group aged more than 50 years. Two patients with the deleterious mutation *BRCA2* or *RAD50* had male family members with breast cancer. There was no significant difference in the incidence of mutations between patients with a family history of female breast/ovarian cancer and early-onset breast cancer patients without a family history (23.1% *vs*. 20.7%, *Pearson's Chi*-*squared test p* = 0.786). However, a significantly higher incidence of deleterious mutations was found in patients with a family history of prostate cancer (*p* = 0.024) or male breast cancer (*p* = 0.008) compared to those without such a family history.

When assessed by the molecular subtype of breast cancer, the mutation prevalence was 19.0% in patients with hormone receptor(HR, +)Her2(−) breast cancer, 20.0% in patients with HR(+)Her2(+) breast cancer, 0% in patients with only Her2(+) cancer, and 45.5% with TNBC (*p* = 0.024). Seven of the 9 *BRCA1* mutations and the single *FANCI* mutation were in the TNBC group; 9 of the 11 *BRCA2*, 1 of the 2 *RAD50* as well as the *BRIP1*, *MSH2*, *MUTYH*, and *RAD51C* mutations were in the hormone receptor (HR)(+)Her2(−) group, and the other *RAD50*, *ATM*, and *TP53* mutations were in the HR(+)Her2(+) group.

### Clinical relevance of the genetic results

Given the clinical significance of the pathogenic variants, patients carrying these pathogenic mutations are considered as high risk to develop cancer. Not only for *BRCA* mutation carriers, but we also provided genetic counseling for carriers of other 10 actionable mutations according to the NCCN guidelines and their cancer risk [15] (Supplementary Table 1).

One TNBC patient, who had received right breast conserving surgery 9 years previously, was referred for genetic tests due to developing left site metachronous breast cancer, which confirmed her germline *BRCA1* mutation, so she decided to undergo bilateral mastectomy with reconstruction and one occult malignancy was found in the resected right breast tissue. In patients with a *BRCA* mutation, one received prophylactic contralateral mastectomy, two decided to undergo prophylactic oophorectomy, and all other *BRCA* mutation carriers preferred not to undergo prophylactic surgery and were advised to have an annual mammography with MRI of the breasts and transvaginal examination and the CA-125 test for prevention of gynecologic malignancy. Two patients with a *TP53* mutation diagnosed as Li-Fraumeni syndrome were advised to have an annual mammography and a comprehensive whole body physical examination. Patients with the pathogenic mutation of *MSH2* and *MUTYH* were advised to consider annual colonoscopy examination for patients and family members. The participant who had breast cancer carrying *MUTYH* mutation underwent colonoscopy, identifying five tubular adenomas that were removed. All patients with deleterious variants involving double-strand DNA repair (*ATM*, *BRIP1*, *FANCI*, *RAD50*, and *RAD51C*) were advised to have an annual screening of the breasts. In addition to the patients themselves, their family members were suggested to test whether they carrying deleterious mutations and mutation carriers were advised to receive screening.

## DISCUSSION

Our study demonstrates a high value of a large gene panel for cancer-risk assessment using the NGS and is the first report of the use of this technique in the Asian-Pacific region. We found 30 (22.6%) pathogenic variants; 9 in *BRCA1*, 11 in *BRCA2*, and 9 in other genes. The mutation prevalence of *BRCA1/2* (15.0%) in patients (Han Chinese) with early-onset or with a significant family history was similar to that reported in Western countries [16] and we found a 7.5% mutation rate of non-*BRCA* genes in women who tested negative for *BRCA1/2* mutation. These data show that multiple gene sequencing increases the mutation detection rate compared to *BRCA* testing alone and that there is no ethnic difference in its application.

In this study, multiple gene testing identify 10 non-*BRCA* mutation carriers. This result is compatible with the suggestion from NCCN guideline that multiple gene sequencing may be more efficient and cost-effective for cancer-risk assessment for patients with a high probability of hereditary breast cancer [15]. Participants found to carry deleterious mutation are considered as high-risk cases to develop malignancy and targeted organ screening are advised for reducing cancer related-mortality. However, the cancer penetrance of non-*BRCA* genes may be intermediate, and there are no standardized screening guidelines. To manage the potential actionable mutations, we provide suggestions based on the biologic functions of these genes. For example, since mutations of *ATM*, *BRIP1*, *FANCI*, *RAD50*, and *RAD51C* affect double-strand DNA repair and may have a similar carcinogenic effect to *BRCA* genes [17, 18], we suggest screening should be started for these carriers. This would provide valuable information about screening for non-*BRCA* mutations and help with future genetic counseling and provide a rationale for a prospective study to elucidate the effect of this policy.

There are still unanswered questions about clinical multiple gene sequencing, such as the design of the gene panel and VUS interpretation. First, it is uncertain how many genes need to survey for testing hereditary breast cancer syndromes so that we do not know if this panel is suitable. The concept of the panel design was that gene mutations in the homologous recombination pathway may have a similar carcinogenic effect to *BRCA* mutations. A recent large study which investigated 17 breast cancer susceptibility genes in 1824 TNBC patients confirmed this rationale, as it showed deleterious mutations in 14 genes, mainly in genes involved in homologous recombination [19]. In addition, mutations in genes for other DNA repair pathway proteins, such as *MSH2* (mismatch DNA repair) and *XRCC1* (base excision repair) are reported to increase breast cancer risk [20, 21]. Germline mutation of tumor suppressor genes, for example *TP53* and *PTEN*, causes hereditary cancers, including breast cancer [22]. Of the 68 genes selected for our panel, 8 were found to be deleterious mutations of non-*BRCA* predisposing genes. A previous large scale study evaluated 42 cancer predisposing genes in 198 patients who met the criteria for *BRCA* testing and found 16 pathogenic variants in 9 non-*BRCA* genes [23]. Combining the results of the above two studies and our own, deleterious mutations have been found in 21 non-*BRCA* genes (Supplementary Table 2). This suggests more studies are warranted to evaluate the selection of predisposing genes for clinical patients.

The increased numbers of VUS identified by multiple gene sequencing is another problem because they cause difficulty in risk assessment and may prompt anxiety and overtreatment for patients. It is therefore important to establish a rapid and robust method for reducing the number of VUS in clinical practice. It is efficient to use bioinformatics analysis to pre-screen the VUS to exclude obvious non-deleterious VUS and select possible deleterious VUSs for functional evaluation [24]. Potential deleterious VUS were selected by mutation frequency analysis (less than 1% in the general population) and a high score using mathematical prediction software (Polyphen2, SIFT and GVGD align). We also performed structural analysis to view whether the mutation affected the protein function. This strategy efficiently reduced the number of VUS, and only 12 strongly suspected of being deleterious were identified in the 68 sequenced genes. However, the result of bioinformatics analysis cannot be used in clinical diagnosis. In order to ensure that the uncertainty did not cause excessive anxiety for these patients, while, at the same time, informing them of the possible risk, they were well-informed and further functional assays are planned. With widespread use of multiple gene sequencing and the sharing of results in an open database (such dbSNP/clinvar at NCBI), the incidence of VUS will decline.

In summary, the 22% prevalence of mutations of cancer predisposing genes is a strong incentive to perform gene testing in these high-risk patients in early cancer screening. We demonstrates that multiple gene sequencing using the NGS is clinically applicable and is an effective method to increase detected rate of high-risk cases, rather than simply testing for *BRCA1/2*. Adequate targeted organ screening may help them to reduce the cancer-related mortality. However, a suitable guide for genetic counseling and better VUS interpretation of non-*BRCA* genes are needed.

## PATIENTS AND METHODS

### Patients

Patients had early-onset breast cancer or bilateral breast cancer or had a family history of breast or ovarian cancer. All patients had to meet one of the following criteria: (1) Early-onset breast cancer (age ≤ 35 years) or bilateral breast cancer; (2) Breast cancer onset age ≤ 50 years and at least one first or second-degree relative with breast cancer or ovarian cancer; (3) Breast cancer onset after the age of 50 years, but with two relatives with breast cancer or one with ovarian cancer [24]. The study was approved by the Institutional Review Board of the National Taiwan University Hospital (201308077RINA).

### Designing of the gene panel

Most predisposing genes in hereditary cancer syndromes are tumor suppressor genes and DNA repair genes [25]. For example, the molecular mechanism of Lynch syndrome is a genetic defect in mismatch repair genes (*MLH1*, *MLH3*, *MSH2*, *MSH3*, *MSH6*, *PMS1*, *PMS2*, and *EPCAM*) [26]. Pathogenic mutations of *BRCA1*, *BRCA2*, and *PALB2*, which involved in homologous recombination for double-strand DNA repair, are linked to hereditary breast cancer, ovarian cancer, and prostate cancer [17]. Defects of the nucleotide excision repair genes cause xeroderma pigmentosum and predispose to skin cancer and lung cancer [27]. Mutations of the *PTEN* gene, a tumor suppressor gene, cause Cowden syndrome and predispose to breast cancer, follicular thyroid cancer, and endometrial cancer [22]. Following a literature review, we hypothesized that germline mutations of DNA repair genes and tumor suppressor genes might predispose to development of breast cancer. Based on this hypothesis, we selected 68 genes for the sequencing panel; these consisted of (i) DNA repair genes involved in homologous recombination, base excision repair, nucleotide excision repair, mismatch repair, nonhomologous DNA end joining, and translesion DNA synthesis; (ii) tumor suppressor genes, such as *PTEN* and *TP53*; and (iii) other genes predisposing to cancer development (Table 1). The overall region of the 68 gene was 4967005 bp (Supplementary Table 3).

### Library preparation, NGS, and sequence mapping

After the patient had given written informed consent, genomic DNA (gDNA) was isolated from peripheral blood mononuclear cells using *QIAGEN Genomic DNA extraction kits (Qiagen* Inc., Valencia, *CA USA) and its* purity and concentration checked by agarose gel electrophoresis and the OD_260/280_ ratio, followed by Covaris fragmentation (Covaris, Inc., Woburn, MA, USA) and checking of the size of the fragmented gDNA using an Agilent Bioanalyzer 2100 (Agilent Technologies, Inc., Santa Clara, CA, USA) and a NanoDrop spectrophotometer (Thermo Fisher Scientific, Inc., Wilmington, DE, USA). Finally, the target gene library was generated using NimblGen capture kits (Roche NimblGen, INC.). The samples were then sequenced on an Illumina MiSeq that generated paired-end reads of 300 nucleotides.

The analysis algorithm is shown as Supplementary Figure 1. The raw sequencing data was aligned with the reference human genome (Feb. 2009, GRCh37/hg19) using Burrows-Wheeler Aligner software (version 0.5.9) [28]. SAMtools (version 0.1.18) was used to perform the necessary data conversion, sorting, and indexing [29]. For single nucleotide polymorphisms (SNPs) and small insertion/deletions (indels), Genome Analysis Toolkit (GATK; version 2.7) was used for variant calling by using Base/indel-calibrator and HaplotypeCaller. Genetic variants larger than 100 bp cannot be identified by GATK, so Pindel or Breakdancer software were used to find structural variants, such as large deletions, insertions, and duplications [30]. After variant calling, ANNOVAR was used for annotation of the genetic variants [31, 32]. Filtering of common variants of sequencing results was performed using dbSNP (version138), Exome sequencing Project 6500 (ESP6500), and the 1000 Genomes variant dataset (2014 Sep). Finally, all potential genetic variants identified in patients were confirmed by repeated PCR amplification of the indicated gene region(s) and direct Sanger sequencing.

In order to check the sensitivity and specificity of the NGS platform and bioinformatics algorithm, we checked the concordance of the results with prior 10 clinical sequencing, which contained large scale deletion and known *BRCA1* genetic variants. The NGS results were fully concordant with the previous sequencing results (Supplementary Methods and Supplementary Figures 1–3).

### Variant classification

The sequence variants were classified according to the IARC variant classification [33]. Large-scale deletion, frame-shift mutation, nonsense mutation, genetic variants associated with uncorrected splicing, and mutations affecting protein function demonstrated by functional analyses are considered as deleterious or pathogenic mutations. An allele frequency greater than 0.01 in the general population in the 1000 Genomes variant dataset (2014Sep) or ESP6500 database suggests a benign or likely benign genetic variant. Silent and intronic variants that do not affect splicing are also considered as benign or probably benign. Other variants, mainly missense mutations without known functional data, are considered to be VUS.

In order to reduce the number of VUS, we used the bioinformatics analysis to evaluate the potential pathogenicity, including PolyPhen2 [34], SIFT [35], and Align GVGD [36], as well as structural analysis. The mathematical prediction is mainly based on an evolutional approach examining the degree of cross-species amino acid conservation by sequence alignment and the properties of the amino acids. After the bioinformatics analysis, we defined VUSs that were suspected of being deleterious mutations as those that met the following two criteria: (1) a population frequency of less than 0.01 in the 1000 Genomes and ESP6500 databases and (2) a bioinformatics analysis result with a SIFT score less than 0.05 and a polyphen2 score greater than 0.95. Several variants were also analyzed using align GVGD software, the results had to be C65 (most likely to interfere with function).

For comparative structural modeling, the variant was simulated based on a known protein structure in the RCSB protein database (http://www.rcsb.org) [24]. For example, a BRCA1 mutant can be created using the SWISS-MODEL program based on the template of the human wild-type BRCA1 BRCT domain interacting with a BACH1 phosphorylated peptide (PDB code: 1T29) [37]. The 3-dimensional structure of the mutation was constructed using the UCSF Chimera program [38].

### Statistical analysis

Descriptive statistics included medians, means, and standard deviations for continuous data. The *X*^2^ test and Fisher's exact test were used to calculate the significance of differences between the means for two groups. All *p* values were 2-sided and *p* values less than 0.05 were considered significant.

## SUPPLEMENTARY MATERIALS FIGURES AND TABLES


