# Supplementary material for: Multiple gene sequencing for risk assessment in patients with early-onset or familial breast cancer
Source: Oncotarget. 2016 Jan 27;7(7):8310–20. doi: 10.18632/oncotarget.7027 (PMC4884994; doi:10.18632/oncotarget.7027)
Supplement: Supplementary file 1 [file oncotarget-07-8310-s001.pdf]

Multiple gene sequencing for risk assessment in patients with early-onset or familial breast cancer

Supplementary Materials

Supplementary Table S1: Summary of suggestions for patients with deleterious mutations in this cohort

Gene	Cancer screening policy	Accordance
BRCA1, *BRCA2*	Annual mammography with MRI for breasts; annual transvaginal examination with CA-125 test for gynecologic malignancy	NCCN guideline
TP53	Annual mammography with MRI for breasts; Comprehensive whole body physical examination	NCCN guideline
MSH2	Annual colposcopy examination	NCCN guideline
MUTYH	Annual colposcopy examination	NCCN guideline
ATM, BRIP1, FANCI, RAD50, and *RAD51C*	Annual mammography with MRI for breasts; annual transvaginal examination with CA-125 test for gynecologic malignancy	Involving in double-strand DNA repair

Reference

NCCN Clinical Practice Guidelines in Oncology. Genetic/familial high-risk assessment: breast and ovarian, version 2.2014.
NCCN Clinical Practice Guidelines in Oncology. Genetic/familial high-risk assessment: colorectal, version 2.2014.

Supplementary Table S2: Deleterious mutations on non-BRCA genes in the three studies

Kurian AW et al.	Couch FJ et al.	NTUH	Combination of three studies
ATM	*ATM*	*ATM*	*ATM*
BLM	*BARD1*	*BRIP1*	*BARD1*
CDH1	*BRIP1*	*FANCI*	*BLM*
CDKN2A	*MRE11A*	*MSH2*	*BRIP1*
MLH1	*NBN*	*MUTYH*	*CDH1*
MUTYH	*PALB2*	*RAD50*	*CDKN2A*
NBN	*PTEN*	*RAD51C*	*FANCI*
PRSS1	*RAD50*	*TP53*	*MLH1*
SXL4	*RAD51C*		*MRE11A*
	RAD51D		*MSH2*
	TP53		*MUTYH*
	XRCC2		*NBN*
			PALB2
			PRSS1
			PTEN
			RAD50
			RAD 51C
			RAD51D
			SXL4
			TP53
			XRCC2

Supplementary Table S3: Genomic regions for targeted genes (hg19, GRCh37)

Gene	chr	start	stop	size
APC	chr5	112038202	112183936	145735
ARLTS1	chr13	50197435	50210008	12574
ATM	chr11	108088559	108241826	153268
BACH1	chr21	30666219	30736217	69999
BARD1	chr2	215588370	215679428	91059
BMPR1A	chr10	88511395	88686945	175551
BRCA1	chr17	41194312	41282387	88076
BRCA2	chr13	32884616	32975809	91194
BRIP1	chr17	59754546	59945920	191375
CDH1	chr16	68737225	68835548	98323
CHEK2	chr22	29081731	29143410	61680
DDB1	chr11	61064918	61115047	50130
DDB2	chr11	47231492	47262769	31278
EPCAM	chr2	47567297	47616167	48871
ERCC1	chr19	45908590	45987086	78497
ERCC2	chr19	45852648	45878845	26198
ERCC3	chr2	128012865	128056752	43888
ERCC4	chr16	14009013	14048205	39193
ERCC5	chr13	103493190	103530351	37162
ERCC6	chr10	50660525	50752169	91645
ERCC8	chr5	60167658	60245905	78248
FANCA	chr16	89801958	89888065	86108
FANCB	chrX	14859528	14896184	36657
FANCC	chr9	97859335	98084991	225657
FANCD2	chr3	10063112	10145614	82503
FANCE	chr6	35415137	35436881	21745
FANCF	chr11	22642078	22652387	10310
FANCG	chr9	35071834	35085013	13180
FANCI	chr15	89782193	89862362	80170
FANCL	chr2	58384377	58473515	89139
FANCM	chr14	45600135	45672093	71959
FANCN (PALB2)	chr16	23612488	23657631	45144
FANCO (RAD51C)	chr17	56764934	56813703	48770
FANCP (SLX4)	chr16	3629182	3666599	37418
FGFR2	chr10	123235848	123362972	127125
GT198/PSMC3IP	chr17	40722328	40734849	12522
MAP3K1	chr5	56105900	56193979	88080
MDM4	chr1	204488051	204544871	64361
MLH1	chr3	37029823	37094409	64587
MLH3	chr14	75478466	75523235	44770
MRE11	chr11	94150895	94232074	81180
MSH2	chr2	47625108	47712367	87260
MSH3	chr5	79945466	80174634	229169
MSH6	chr2	48005221	48036092	30872

MYH (MUTYH)	chr1	45792914	45811142	18229
NBN	chr8	90943564	91001944	58381
OGG1	chr3	9786627	9810353	23727
PMS1	chr2	190643811	190744355	100545
PMS2	chr7	6010870	6053737	42868
POLB	chr8	42190973	42234331	43359
POLD1	chr19	50882579	50923275	40697
POLE	chr12	133198347	133268945	70599
POLH	chr6	43538877	43590260	51384
POLK	chr5	74802656	74897646	94991
PTEN	chr10	89617870	89733687	115818
RAD50	chr5	131886711	131982313	95603
RAD51	chr15	40981972	41026354	44383
RAD51D	chr17	33424810	33453541	28732
SMAD4	chr18	48489386	48613411	124026
STK11	chr19	1200740	1230428	29689
TP53	chr17	7563097	7595856	32760
XPA	chr9	100435190	100464691	29502
XPC	chr3	14184647	14225172	40526
XRCC2	chr7	152341587	152378250	36664
XRCC3	chr14	104161953	104186823	24871
XRCC4	chr5	82368316	82651579	283264
XRCC5	chr2	216969019	217073016	103998
XRCC6	chr22	42012294	42062052	49759

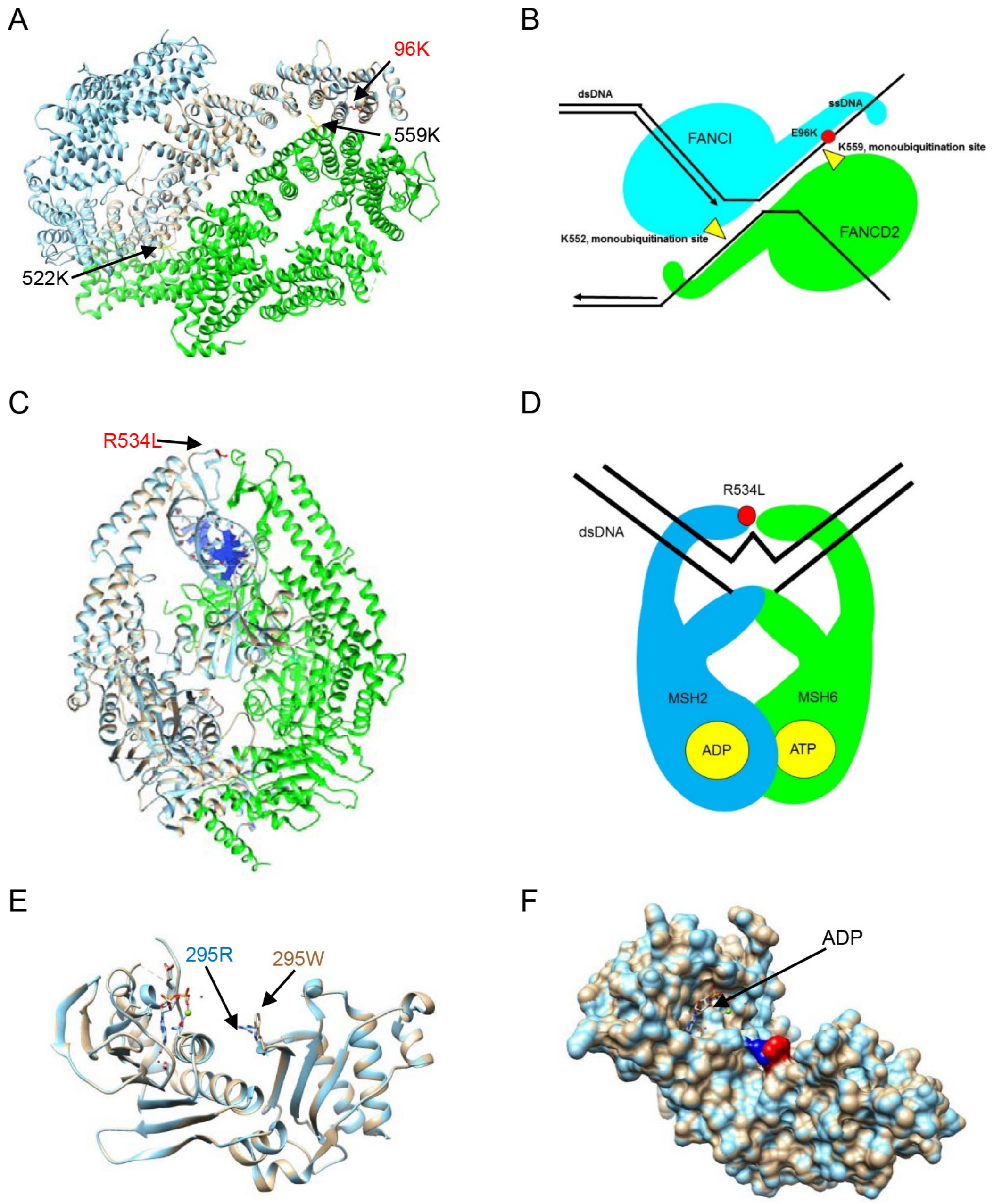

Supplementary Figure S1: Structure analysis of three mutations. (**A**) Ribbon presentation and (**B**) cartoon picture of the FANCI E96K interacting with FACD2 (green color). FANCI E96K mutant protein (khaki color) is superimposed with wild type FANCI (light blue color). (**C**) Ribbon diaphragm of MSH2 R534L (Khaki color) superimposed with wild type MSH2 (light blue color) and (**D**) cartoon picture showing missense mutation R534L located at the clamping region, involved in DNA contact, ATP hydrolysis and matching of MSH6 (green color). (**E**) Structure of mutant PMS2 Trp295 (khaki color) imposed with wild type PMS2 (light blue color). (**F**) Surface view of ADP binding pocket and the 3-dimentional location of the Arg295 (dark blue) and Trp295 (red).

Supplement method Post-next generation sequencing (NGS) bioinformatics analysis

The analysis algorithm is illustrated as supplement figure 2. The Fastq files generated form illumine Miseq were aligned to the reference human genome (Feb. 2009, GRCh37/hg19) using the Burrows-Wheeler Aligner (BWA) software (version 0.5.9) [1]. The SAMtools (version 0.1.18) was used to perform the necessary data conversion, sorting, and indexing [2]. For single nucleotide polymorphism (SNP) and small insertion/ deletions (indel), Genome Analysis Toolkit (GATK; version 2.7) was used for variants calling. When genetic variants larger than 100 bp, GATK cannot identify them so as to Pindel or Breakdancer soft wares instead. Pindel and Breakdancer can find structural variants such as large deletion, insertion and duplication. After variants calling, ANNOVAR was used for annotation of the genetic variants [3, 4]. Filtering of common variants of sequencing results was performed using dbSNP (version138), Exome sequencing Project 6500 (ESP6500), the 1000 Genomes variant dataset (2014Sep).

In order to confirm this algorithm worked, we pretested 10 samples with known results of *BRCA1* or *MLH1* [5, 6]. Eight *BRCA1* samples are from previous *BRCA* study, one is cell line HCC1937 DNA and the *MLH1* sample is kindly provided from Dr. Wei [5, 6]. Among eight patient samples of *BRCA1*, there were only benign SNP found in the previous study, which preformed sequencing in *BRCA1* exons and exon-intron boundaries. The genetic variants found in the previous study can be 100% identified by NGS method and analysis. For HCC1937 cell line, which contains *BRCA1−/−*, is detected in the NGS run and called by GATK program, illustrated in the supplementary figure 3. The large deletion of *MLH1* is not found in the GATK but identified by Pindel/ Breakdancer (supplementary figure 4). The deleted region can be visualized in the IGV and break point is clearly demonstrated in the Pindel result.

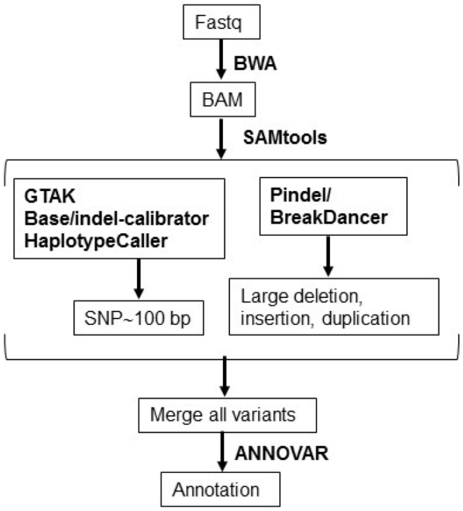

Supplementary Figure S2: Algorithm of the post-NGS analysis.

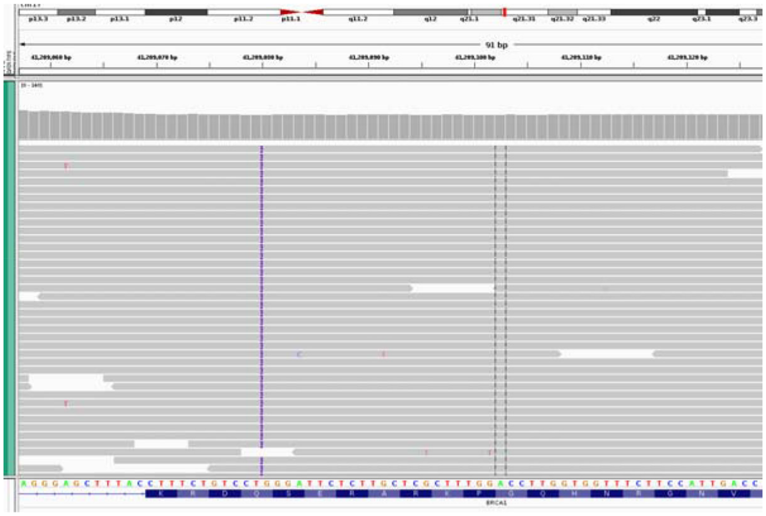

Supplementary Figure S3: *BRCA1* NM_007300.3:c.dupC (homozygous), illustrated by IGV, the purple colour column represents the insertion C.

```
16      D 5286  NT 0 "" ChrID chr3      BP 37084589     37089876        BP_range 37084589       37089877        Supports 6      6       + 3     3
GAGTTAAAAATCATACTACCTGTTTATATGGTTTCATATCCACTTTTTTCATGTGATATActcta<5276>cccagCCCAATCAAGTAACGTGGTCACCCAGAGTGGCAGATAGGAGCACAAGGCCTGGGAAAGCACTGGAGAAAT
                                         ACTTTTTTCATGTGATATA                CCCAATCAAGTAACGTGGTCACCCAGAGTGGCAGATAGGAGCACAAGGCCTGGGAAAGCACTGGAGAAAT
                                         ACTTTTTTCATGTGATATA                CCCAATCAAGTAACGTGGTCACCCAGAGTGGCAGATAGGAGCACAAGGCCTGGGAAAGCACTGGAGAAAT
                                    TATCCACTTTTTTCATGTGATATA                CCCAATCAAGTAACGTGGTCACCCAGAGTGGCAGATAGGAGCACAAGGCCTGGGAAAGCACTGGAGAAAT
GAGTTAAAAATCATACTACCTGTTTATATGGTTTCATATCCACTTTTTTCATGTGATATA                CCCAATCAAGTAACGTGGTCACCCAGAGTGGCAGATAGGAGCACAAGGCCTGGGAAAGCACTGGAGAAAT
GAGTTAAAAATCATACTACCTGTTTATATGGTTTCATATCCACTTTTTTCATGTGATATA                CCCAATCAAGTAACGTGGTCACCCAGAGTGGCA
GAGTTAAAAATCATACTACCTGTTTATATGGTTTCATATCCACTTTTTTCATGTGATATA                CCCAATCAAGTAACGTGGTCACCCAGAG
```

IGV

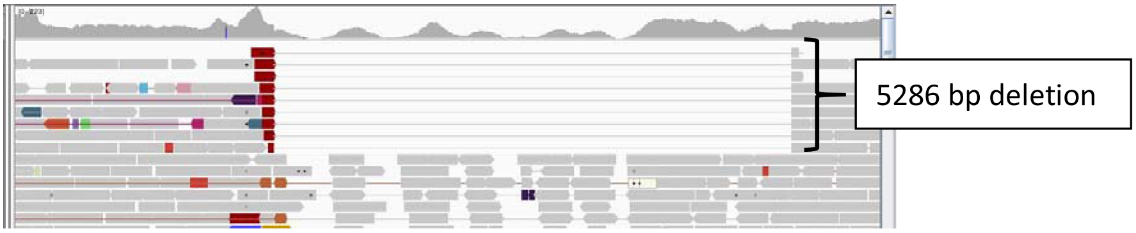

Sanger sequencing

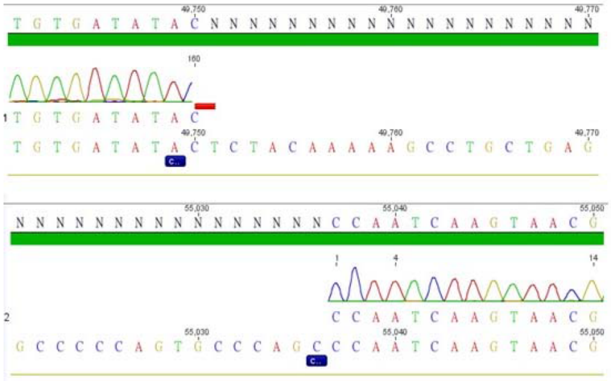

Supplementary Figure S4: *MLH1* deleted region: chromosome 3, 37084589–37089876 (heterozygous)

REFERENCES

1. Li H, Durbin R. Fast and accurate short read alignment with Burrows¬Wheeler transform. Bioinformatics. 2009; 25:1754–1760.
2. Li H, Handsaker B, Wysoker A, Fennell T, Ruan J, Homer N, Marth G, Abecasis G, Durbin R, Genome Project Data Processing S. The Sequence Alignment/Map format and SAMtools. Bioinformatics. 2009; 25:2078–2079.
3. McKenna A, Hanna M, Banks E, Sivachenko A, Cibulskis K, Kernytsky A, Garimella K, Altshuler D, Gabriel S, Daly M, DePristo MA. The Genome Analysis Toolkit: a MapReduce framework for analyzing next-generation DNA sequencing data. Genome research. 2010; 20:1297–1303.
4. Wang K, Li M, Hakonarson H. ANNOVAR: functional annotation of genetic variants from high-throughput sequencing data. Nucleic acids research. 2010; 38:e164.
5. Kuo WH, Lin PH, Huang AC, Chien YH, Liu TP, Lu YS, Bai LY, Sargeant AM, Lin CH, Cheng AL, Hsieh FJ, Hwu WL, Chang KJ. Multimodel assessment of BRCA1 mutations in Taiwanese (ethnic Chinese) women with early-onset, bilateral or familial breast cancer. Journal of human genetics. 2012; 57:130–138.
6. Wei SC, Yu CY, Tsai-Wu JJ, Su YN, Sheu JC, Wu CH, Wang CY and Wong JM. Low mutation rate of hMSH2 and hMLH1 in Taiwanese hereditary non-polyposis colorectal cancer. Clin Genet. 2003; 64:243–251.
